# A predation assay using amoebae to screen for virulence factors unearthed the first *W. chondrophila* inclusion membrane protein

**DOI:** 10.1038/s41598-019-55511-1

**Published:** 2019-12-20

**Authors:** C. Kebbi-Beghdadi, L. Pilloux, A. Croxatto, N. Tosetti, T. Pillonel, G. Greub

**Affiliations:** Center for Research on Intracellular Bacteria, Institute of Microbiology, Centre Hospitalier Universitaire Vaudois, Lausanne, Switzerland

**Keywords:** Bacteria, Clinical microbiology

## Abstract

*Waddlia chondrophila* is an intracellular bacterium phylogenetically related to the well-studied human and animal pathogens of the *Chlamydiaceae* family. In the last decade, *W. chondrophila* was convincingly demonstrated to be associated with adverse pregnancy outcomes in humans and abortions in animals. All members of the phylum *Chlamydiae* possess a Type Three Secretion System that they use for delivering virulence proteins into the host cell cytosol to modulate their environment and create optimal conditions to complete their life cycle. To identify *W. chondrophila* virulence proteins, we used an original screening approach that combines a cosmid library with an assay monitoring resistance to predation by phagocytic amoebae. This technique combined with bioinformatic data allowed the identification of 28 candidate virulence proteins, including Wimp1, the first identified inclusion membrane protein of *W. chondrophila*.

## Introduction

*Waddlia chondrophila* is an obligate intracellular bacterium belonging to the phylum *Chlamydiae*. This phylum comprises the *Chlamydiaceae* family including several well-studied human and animal pathogens such as *Chlamydia trachomatis, Chlamydia pneumoniae* and *Chlamydia psittaci* in addition to several other families of *Chlamydia*-related bacteria. A recently published comparative analysis of this phylum, based on genomic data, identified thirteen family level lineages of *Chlamydia*-related bacteria^1^ which have been isolated from mammals, fish or arthropods as well as other environmental samples^2^. Some of them are symbionts of protists while others, such as *Piscichlamydia salmonis*, *Parachlamydia acanthamoebae, Simkania negevensis* or *Waddlia chondrophila*, are emerging animal or human pathogens^2,3^.

*W. chondrophila* was isolated twice from an aborted bovine fetus, once in the USA and once in Germany^4,5^. Since then it has been reported as a bovine abortigenic agent in several studies using both serological and molecular methods^6,7^. In humans, *W. chondrophila* is associated with adverse pregnancy outcomes, tubal infertility or respiratory tract infections^8–13^. Consistent with its pathogenic potential, *W. chondrophila* is able to infect and propagate in human macrophages, the first line of defense against infection, as well as in endometrial cells and pneumocytes^14,15^.

Like all members of the *Chlamydiae* phylum, *W. chondrophila* exhibits a biphasic life cycle with the infectious form, the Elementary Body (EB) entering the host cell and rapidly evading the endocytic pathway to establish a replicative niche in a vacuolar compartment that is named the inclusion. EBs then differentiate into Reticulate Bodies (RBs), the replicative form that divides by binary fission. At the end of the exponential growth phase, RBs redifferentiate into EBs that lyse their host cell and are ready to start a new cycle^16^.

As a strict intracellular organism, *W. chondrophila* closely interacts with its host cell in order to create optimal conditions for the completion of its life cycle. For this purpose, it secretes virulence proteins or effector molecules into the host cell cytoplasm, mainly via its Type 3 Secretion System (T3SS). The T3SS is a syringe-like structure spanning the inner and outer bacterial membranes as well as the inclusion membrane, thereby allowing direct secretion from the bacteria into the host cell cytosol^17,18^. The structural proteins forming the T3SS apparatus, as well as the chaperones required for maintenance of the effectors in a secretion-competent state, are very well conserved between distantly related bacteria encoding similar secretion systems and between all known members of the *Chlamydiae*. Indeed a comparative analysis of *Chlamydiae* T3SS genomic data indicates that genes encoding the structural components and chaperones of T3SS are present in genomes of all members of the phylum^1^. T3SS genes in *Chlamydiae* are split between four different loci, but the genetic organization is conserved, indicating that this secretion system was probably already present in the common ancestor of these bacteria^3,19,20^. Despite the high degree of conservation of T3SS structural components, effector proteins are very poorly conserved between different bacterial species and are largely species-specific. Indeed, only a few T3SS effectors identified in *Chlamydiaceae* have identifiable homologs in *W. chondrophila*^1^. T3SS effectors are usually either found at the bacterial surface or are secreted into the host cell cytoplasm^17^. In addition, some effectors localize to the inclusion membrane, where their position at the interface between the bacteria-containing vacuole and the host cell is ideal to facilitate their manipulation of the host. These T3SS effectors are called inclusion membrane proteins. A subset of these proteins, characterized by a bilobed hydrophobic domain, are named Inc proteins (Incs). Dozens of Incs have been bioinformatically identified or experimentally studied in *Chlamydiaceae* bacteria^21–25^, however only a few of them have been carefully well characterized so far, mainly those of *C. trachomatis*. They mediate a wide range of bacteria-host cell processes, including vesicular trafficking, microtubule modifications, interactions with RAB GTPases and modulation of other host signaling pathways and cellular functions^26–30^. In addition, Incs also play structural roles and are important in enabling homotypic fusion of inclusions or for maintaining the stability of the inclusion membrane^31,32^.

The species specificity of Inc proteins and more generally of T3SS effectors probably reflects the very diverse life styles of *Chlamydiae* bacteria. Indeed, *W. chondrophila* is able to enter and multiply in a broad range of hosts including protists, insect, fish and mammalian cell lines^15,33,34^. Whereas the tropism of *Chlamydiaceae* bacteria is mainly restricted to mammalian cells^35,36^. Furthermore, their trafficking in host cell is different. *W. chondrophila* recruit mitochondria around their replicative vacuole and associate with endoplasmic reticulum^14^ whereas *C. trachomatis* disrupt the host cell Golgi and intercept vesicular traffic to the plasma membrane to obtain lipids^37,38^. The identification and characterization of virulence proteins of *W. chondrophila* will shed light on several important aspects of the pathogenicity of these strictly intracellular bacteria and may help to understand how *W. chondrophila* subvert host cell pathways to their own advantage.

In the present work, we used a novel approach to identify *W. chondrophila* candidate virulence proteins. This approach combines a genomic library in cosmids with a screen using a lysis plaque assay that monitors resistance of cosmid-transduced *E.coli* to predation by phagocytic amoebae^39^. Indeed, several studies with intracellular bacteria have demonstrated that genes required for resistance to predation by amoebae are also required for replication or survival in mammalian phagocytes or even for causing diseases in animals^40^. Utilizing our original and transferable screening approach, combined with bioinformatics analyses, we identified 28 putative *W. chondrophila* virulence proteins. Focusing on one of them, hypothetical protein Wcw_1131, we could highlight its T3SS dependent secretion and characterize its gene and protein expression during the course of an infection as well as its localization within the inclusion. Altogether, these results unveil Wimp1 as the first *W. chondrophila* inclusion membrane protein.

## Results

### Identification of Wcw_1131 as a potential virulence factor of *W. chondrophila*

**Figure 1:**
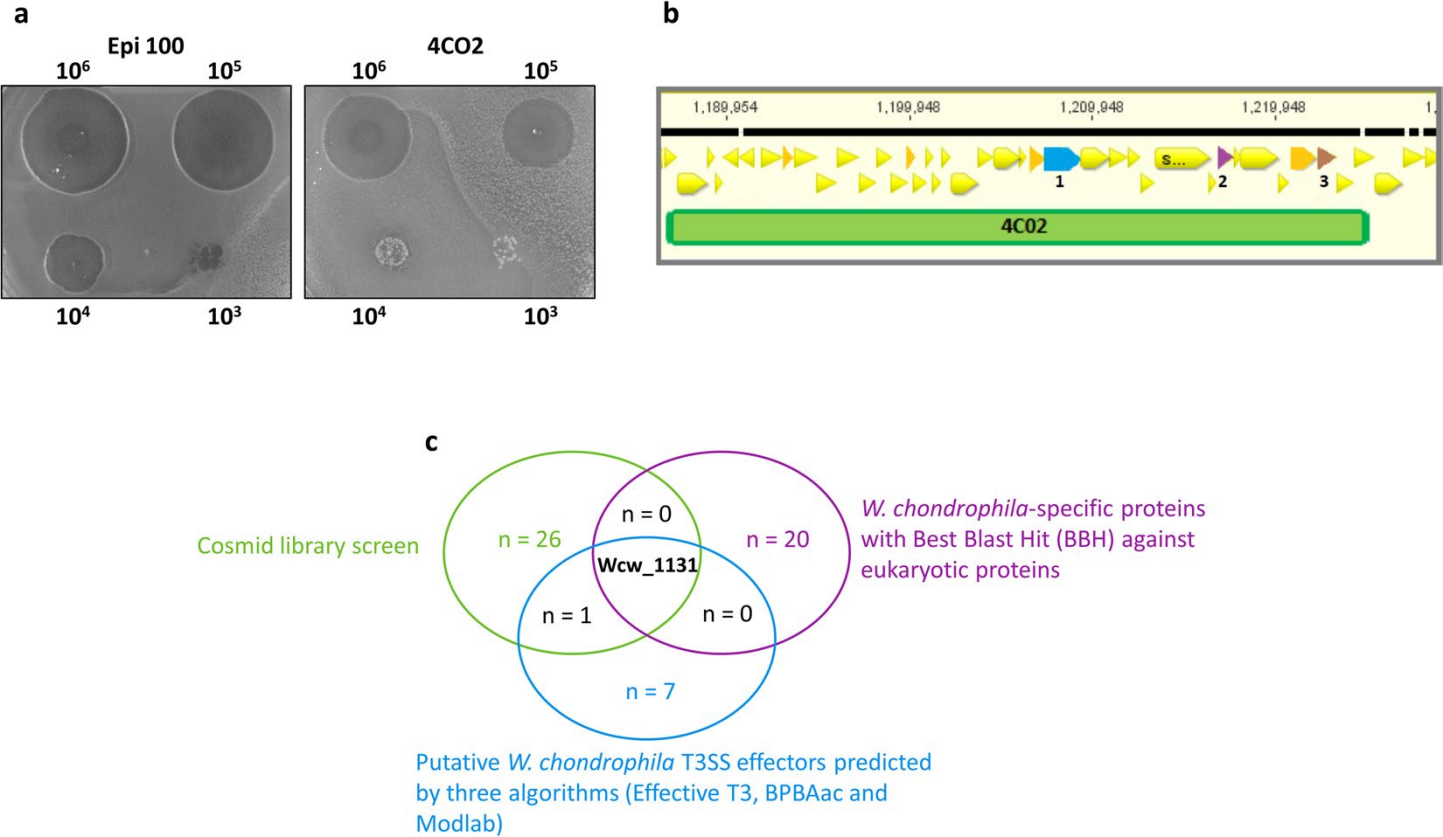
Predation assay using amoebae. **(a)** Resistance of *E.coli* harbouring *W. chondrophila* cosmid 4C02 to predation by amoebae is compared to resistance of *E.coli* containing the empty plasmid (Epi100). Gain of toxicity resulted in smaller lysis plaques. 10^6^, 10^5^, 10^4^, or 10^3^ *A. castellanii* were spread over the bacterial layer. **(b)** Cosmid 4CO2 was sequenced and mapped on the *W. chondrophila* genome. The genomic region covered by this cosmid contains multiple Open Reading frames (ORFs) whose predicted function is not related to virulence (yellow triangles). However, three genes encoding putative virulence proteins were identified in this genomic region and highlighted in color: 1 = wcw_1131; 2 = wcw_1138; 3 = wcw_1143. For detailed analysis of these genes, see Supplementary Table 1. **(c)** Graphical representation of three different screenings of *W. chondrophila* genome for virulence proteins. Hypothetical protein Wcw_1131 is the only protein recovered in the three screenings.

*Acanthamoeba castellanii* are unicellular protists able to feed on *E. coli* and to create large lysis plaques in a bacterial layer. Resistance to predation results in smaller or delayed plaque formation and correlates with virulence^39,41–44^. In order to identify candidate virulence proteins, we performed a lysis plaque assay with *E. coli* transduced with cosmids containing a genomic library of *W. chondrophila*. Amoebae were spread over a layer of *E. coli* harboring different clones of the cosmid library and bacterial resistance to predation was visualized macroscopically and compared to the *E. coli* reference strain (Fig. 1a). In a preliminary screening of 200 cosmids (the complete library contains 1600 cosmids), we observed an increased resistance to predation by *A. castellanii* for 23 cosmids that were sequenced and mapped to the *W. chondrophila* genome^19^ (Fig. 1b). These 23 cosmids of interest were further analyzed for the presence of genes potentially involved in virulence, based on homology to known virulence proteins, predicted domains and presence of predicted transmembrane regions or signal peptides. Twenty-eight candidate virulence proteins were identified using this cosmid library screening (Supplementary Table S1) including Mip (Wcw_0028), a conserved virulence factor encoded on the genome of all members of the *Chlamydiae*, demonstrating the pertinence of this approach to identify virulence proteins of *W. chondrophila*. We also identified putative new virulence proteins, including a protein belonging to an uncharacterized protein family that includes a number of plasmid-encoded virulence proteins (Wcw_0023, Pfam uncharacterized protein family UPF0137), a homolog of the Type IV secreted protein Hcp (Wcw_0936), a hemolysin (Wcw_1828) and a hydrolase (Wcw_1835).

Among the 28 putative virulence proteins listed in Supplementary Table S1, we focused our interest on hypothetical protein Wcw_1131, that exhibits a Ras guanine-nucleotide exchange factors (Ras GEF) domain (PF00617), a eukaryotic domain implicated in the activation of small GTPases such as those of the Ras superfamily^45^. This domain has been described in bacterial virulence proteins^46^ and indicates that Wcw_1131 protein is probably able to interact with eukaryotic host proteins, with a putative role in virulence that needs to be investigated.

### Bioinformatic analyses suggest that Wcw_1131 is a virulence protein secreted by Type III Secretion System

Apart from its Ras GEF domain, hypothetical protein Wcw_1131 does not display any significant feature that could give hints about its function or localization during *W. chondrophila* infection. This 74 kDa protein is strictly specific to *W. chondrophila* and has no identifiable homolog in any other bacteria. According to the Interpro database, the Ras guanine-nucleotide exchange factors catalytic domain (IPR001895) was identified in only 153 bacterial proteins in UniProtKB (versus 17784 eukaryotic proteins). Interestingly, most of these 153 proteins are encoded in the genome of known intracellular bacteria, including *Chlamydiae* spp., *Legionella* spp., *Piscirickettsia salmonis*, one *Coxiella* sp. and two *Berkiella* spp.^47,48^. Wcw_1131 Best Blast Hits (BBH) with the NCBI nr database are against eukaryotic proteins containing a Ras GEF domain (about 30% identity in the Ras GEF domain region). Twenty other *W. chondrophila*-specific proteins exhibit a BBH against eukaryotic proteins (Supplementary Table S2) and are thus likely to be implicated in bacteria-host interactions. In addition, Wcw_1131 protein is predicted *in silico* to be a Type 3 Secretion System effector by three different algorithms: Effective T3, BPBAac and Modlab^49–51^. Supplementary Table S3 shows the nine *W. chondrophila* proteins predicted by these three algorithms to be T3SS effectors. Interestingly, Wcw_1131 is the only protein retrieved in the cosmid screen for virulence proteins that has a BBH against eukaryotic proteins and is predicted to be a T3SS effector by the three algorithms mentioned above (Fig. 1c). It has no predicted signal peptide or transmembrane domain nor does it display the typical hydrophobic bilobed structure of chlamydial Inc proteins^23,25^.

### Wcw_1131 is secreted by Type III Secretion System

As *W. chondrophila* is not amenable to genetic manipulation, we assessed Type 3-dependent secretion of recombinant Wcw_1131 in *Yersinia enterocolitica*, a heterologous system that has, thus far, been successfully used to demonstrate secretion of chlamydial effectors, including inclusion proteins^52–54^. In this assay, *Y. enterocolitica* Type 3 Secretion System is repressed or activated depending on the presence or absence of calcium in the culture medium^55^. Full length Wcw_1131 was cloned in the low copy number plasmid pBAD-DEST49 that allows addition of a V5 epitope to the C-terminal end of the protein. We used immunoblot to look at the presence of Wcw_1131 and of the negative control SecA in bacterial pellets and culture supernatants when the T3SS was activated or not (Supplementary. Fig. S2a). Wcw_1131 was retrieved in the supernatant only when the T3SS was activated. The non-secreted protein SecA was not detected in the supernatant in any of the conditions. Congruent results were obtained in four independent experiments. To exclude the possibility that presence of the protein in the culture supernatant was due to non-specific lysis of the bacteria, cell viability was compared in both conditions by counting CFU. These results were further confirmed when a chemically-synthetized specific T3SS inhibitor^56^ was added to the culture medium in T3SS-activated conditions. Inhibitor addition resulted in a decrease of the amount of protein detected in the supernatant, while the signal intensity in the bacterial pellet did not vary (Supplementary. Fig. S2a). Experiments with the T3SS inhibitor were performed in triplicates and gave similar results. Signal intensities were measured with Image J and the ratio of values detected in supernatant versus pellet fractions were calculated for each tested condition (Supplementary. Fig. S2b). Data obtained in this assay suggested that secretion of Wcw_1131 is dependent on a functional T3SS. However, due to the low number of replicates as well as to the variability between immunoblots, differences between the three conditions were not statistically significant.

**Figure 2:**
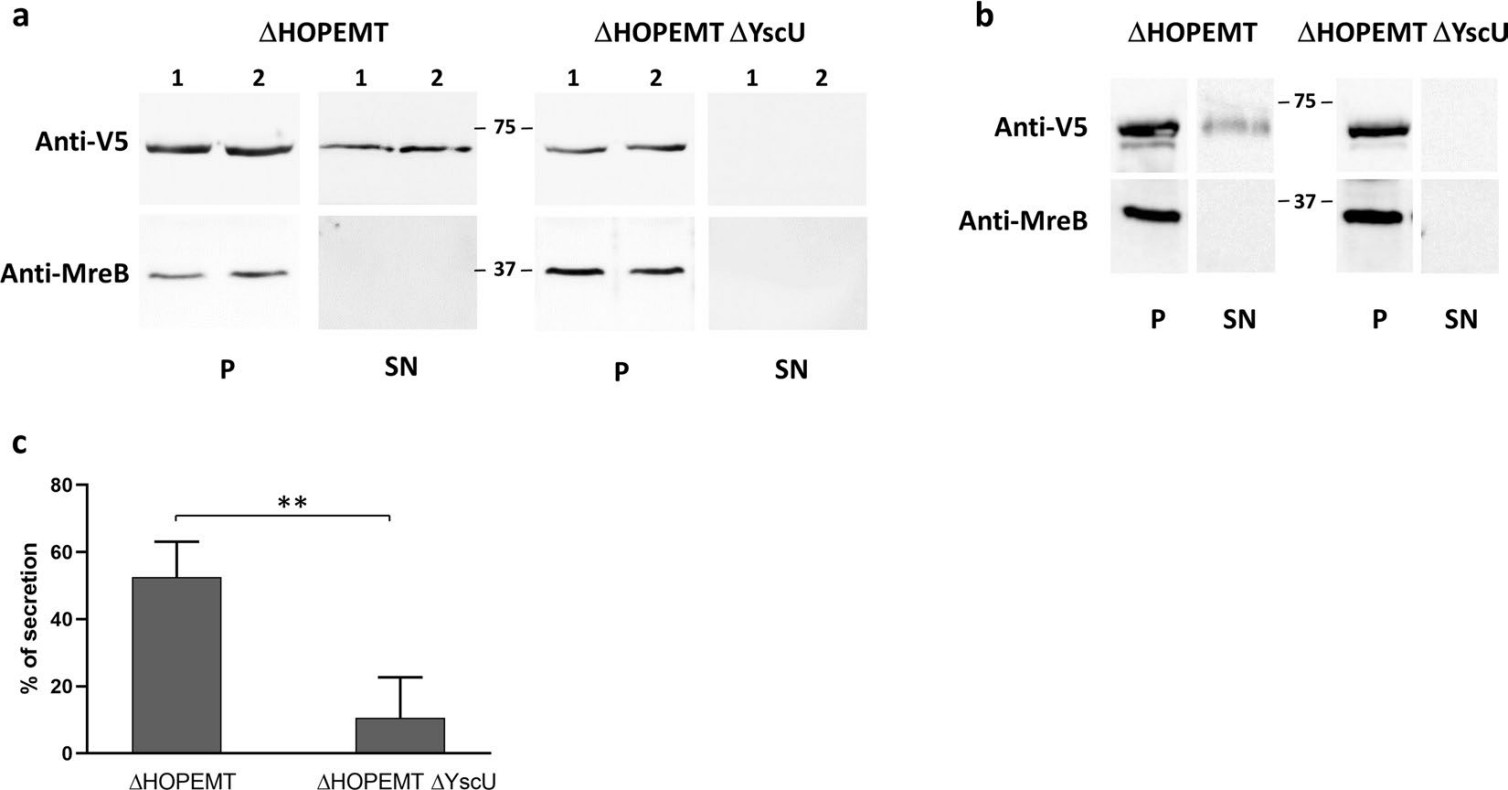
Type 3-dependent secretion of Wcw_1131 in *Y. enterocolitica*. **(a)** Wcw_1131 tagged with a V5 epitope was detected by immunoblot in the bacterial pellet (P) or in the culture supernatant (SN) of *Y. enterocolitica* T3S-proficient (ΔHOPEMT) and T3S-deficient (ΔHOPEMT ΔYscU) strains. Two out of 6 independent experiments are displayed in this figure. Full-length immunoblots are presented in Supplementary Fig. S1. MreB is a strictly cytosolic *Y. enterocolitica* protein. Its absence of detection in the supernatant fractions demonstrates that the presence of Wcw_1131 in these same fractions does not result from bacterial lysis or contamination. **(b)** Same assay performed with the positive control *C. trachomatis* TepP. Full-length immunoblots are presented in Supplementary Fig. S1. **(c)** Signal intensities were measured with Image J software and the percentage of Wcw_1131 secretion by *Y. enterocolitica* ΔHOPEMT or ΔHOPEMT ΔYscU was calculated as the ratio between the amount of secreted and total protein. Results are the means and SD of 6 independent experiments. **p = 0.0022.

In order to clarify these results, we used another *Y. enterocolitica* secretion assay that involves T3S-proficient (ΔHOPEMT) and T3S-deficient (ΔHOPEMT ΔYscU) strains^53^. Full length Wcw_1131 tagged with a C-terminal V5 epitope was cloned in the low copy number plasmid pLJM3 where expression of the gene of interest is driven by the promoter of the *Y. enterocolitica YopE* gene^53^. We checked by immunoblot the presence of Wcw_1131 in bacterial pellets and culture supernatants from both *Y. enterocolitica* strains (Fig. 2a). Similar amounts of proteins were detected in the bacterial pellets for both strains, indicating proper expression of the protein. Wcw_1131 was detected in the supernatant of the T3S-proficient strain in all experiments (n = 6). In the supernatant of T3-deficient strain, Wcw_1131 was either absent (n = 3) or weakly detected (n = 3). The *C. trachomatis* T3SS effector TepP (translocated early phosphoprotein) was used as positive control in this assay (Fig. 2b)^57^. Signal intensities on immunoblots were measured with Image J and the percentage of secretion was calculated as the ratio between the amount of protein in the supernatant fraction and the total amount of protein in pellet and supernatant fractions. For Wcw_1131, this percentage was significantly higher in the T3S-proficient strain than in the T3-deficient one (Fig. 2c). The absence of the strictly cytosolic protein MreB in the supernatant fractions demonstrates that the presence of Wcw_1131 in these same fractions does not result from bacterial lysis or contamination.

Altogether, results obtained in *Y. enterocolitica* demonstrated that secretion of Wcw_1131 is dependent on a functional T3SS.

### *wcw_1131* gene is expressed early during the course of a replication cycle

**Figure 3:**
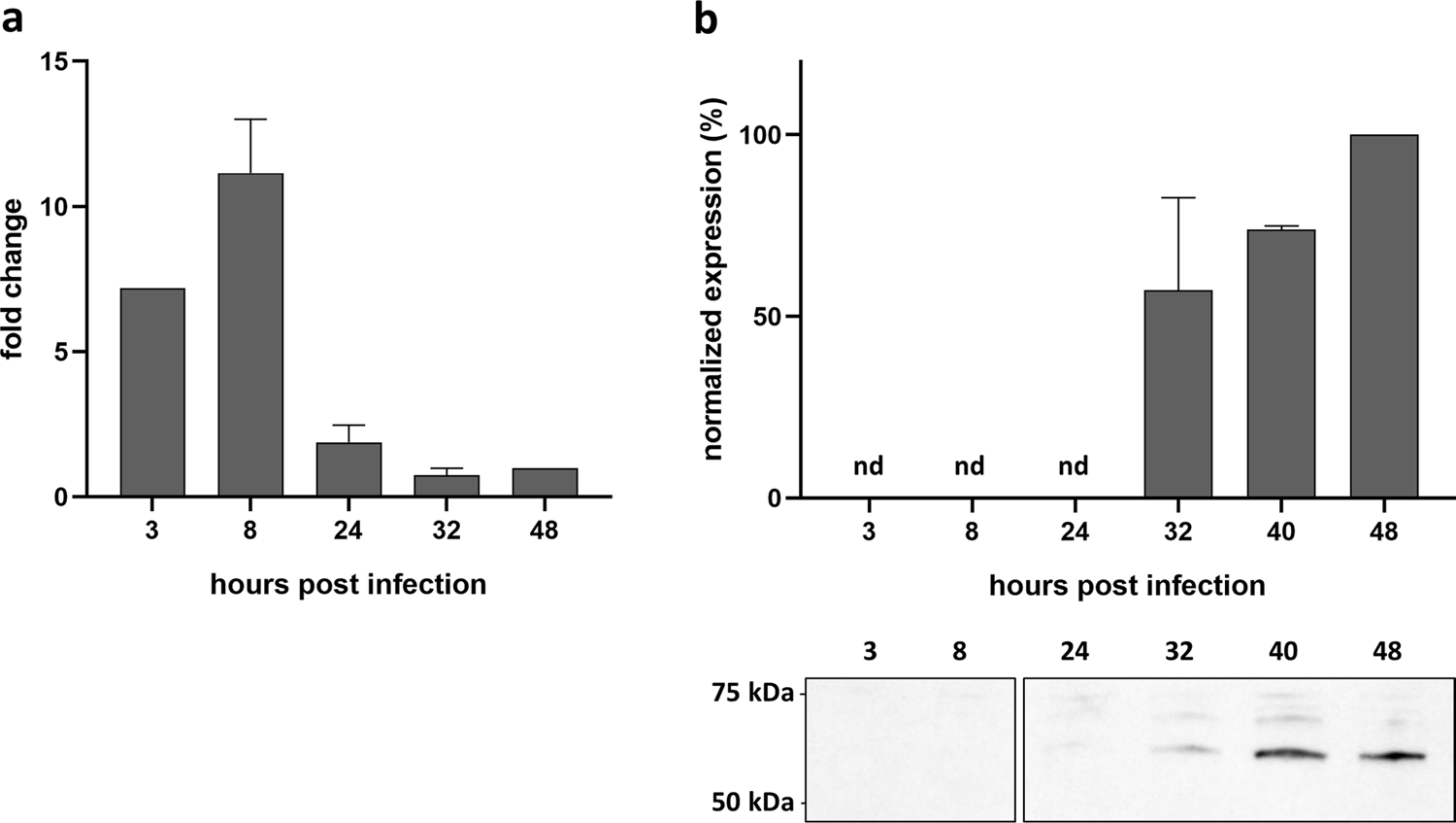
Wcw_1131 is expressed during the early/mid phases of the *W. chondrophila* life cycle. *wcw_1131* transcriptional expression was analysed by qRTPCR during the course of an infection in Vero cells and normalized at 48 h pi according to 16SrRNA gene expression. Results are the means and SD of three independent experiments. **(b)** Wcw_1131 protein expression was monitored by immunoblot during the course of an infection in Vero cells (full-length immunoblot is presented in Supplementary Fig. S4). The signal intensity measured with ImageJ was normalized according to the number of bacteria in the sample and expressed as percentages of the maximum value. Results are the means and SD of three independent experiments.

The transcriptional pattern of gene *wcw_1131* was determined by qRTPCR at different time points following infection of Vero cells with *W. chondrophila*. In this cell line, EBs differentiate into RBs as early as 3 hours post infection (pi). Exponential multiplication occurs between 8 and 32 hours pi with a doubling time of about 80 min^34^. RBs then asynchronously re-differentiate into EBs that are released by host cell lysis. The replication cycle is completed in about 48 hours^15,58^. qRTPCR results were normalized at 48 hours pi using 16SrRNA gene as an internal reference. The transcription profile indicates that *wcw_1131* gene is transcribed very early during the replication cycle with a peak of RNA detected at 8 hours pi (Fig. 3a).

**Figure 4:**
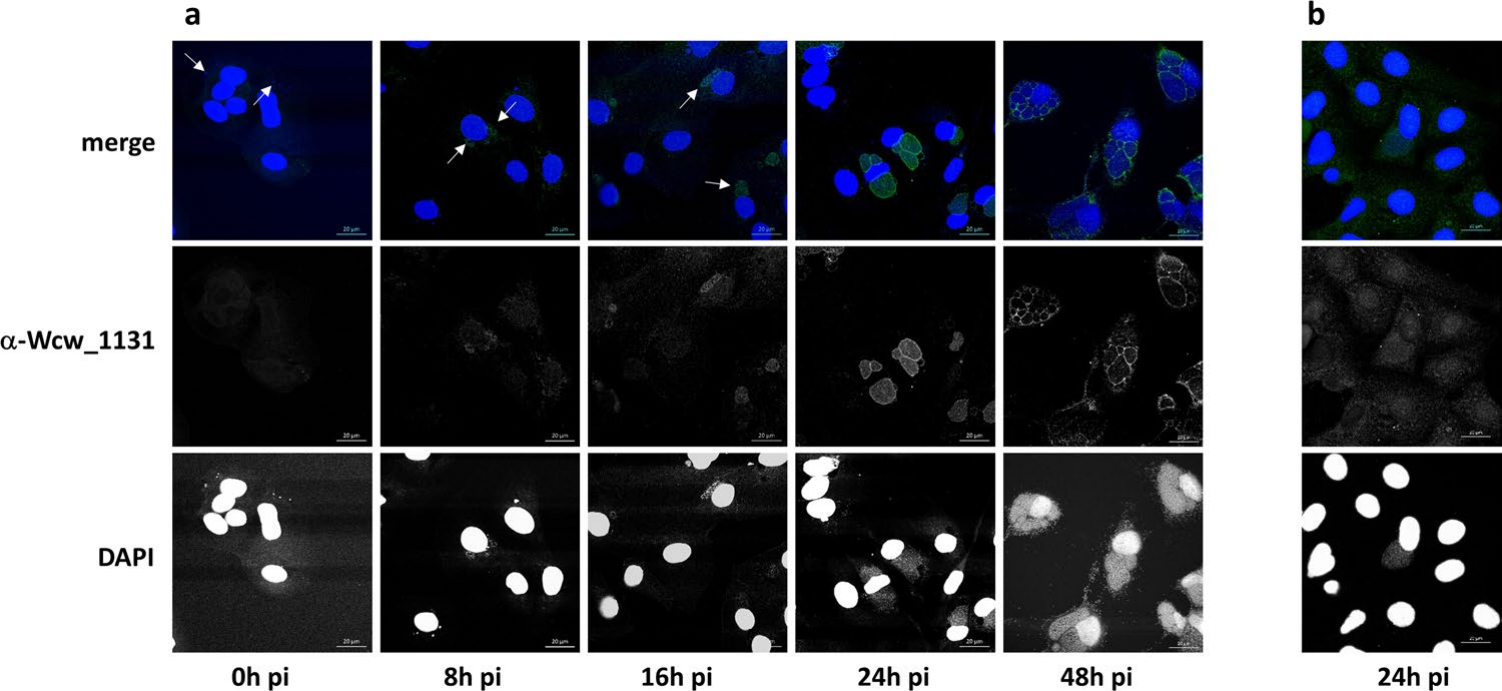
Wcw_1131 localizes at the *W. chondrophila* inclusion membrane during infection. Vero cells infected with *W. chondrophila* were stained with a polyclonal antibody raised against Wcw_1131 (green) and with DAPI (blue) at different time points after infection covering the entire bacterial development cycle. White arrows point at EBs (at 0 h post infection) or at small inclusions (at 8 and 16 hours post infection). **(b)** Vero cells infected with *Estrella lausannensis* during 24 hours were incubated with anti-Wcw_1131 (green) and DAPI (blue). Bar: 20 μm.

Protein expression was analyzed during the course of a replication cycle by immunoblot using a specific anti-Wcw_1131 antibody (Fig. 3b). Unfortunately, no signal could be detected earlier than 32 hours pi probably due to the low abundance of the protein as well as to the weak sensitivity of this technique. Western blot results indicated roughly similar levels of Wcw_1131 at the different time points, with only a two-fold increase at 48 hours pi as compared to 32 hours pi. However, our RNA analyses suggested that *wcw_1131* is an early-transcribed gene and we expected the corresponding protein to be produced during the early phase of the replication cycle. In absence of a detectable signal at time points 3, 8 or 24 hours pi, we could only speculate on the temporal expression of Wcw_1131. This matter was clarified when we performed immunostaining on *W. chondrophila*-infected Vero cells to localize the protein. With this more sensitive technique, we could detect a signal corresponding to protein Wcw_1131 already at 8 hours pi. At this time point, the signal co-localizes with bacteria in small inclusions (Fig. 4).

### Wcw_1131 protein is located within the *W. chondrophila* inclusion membrane

**Figure 5:**
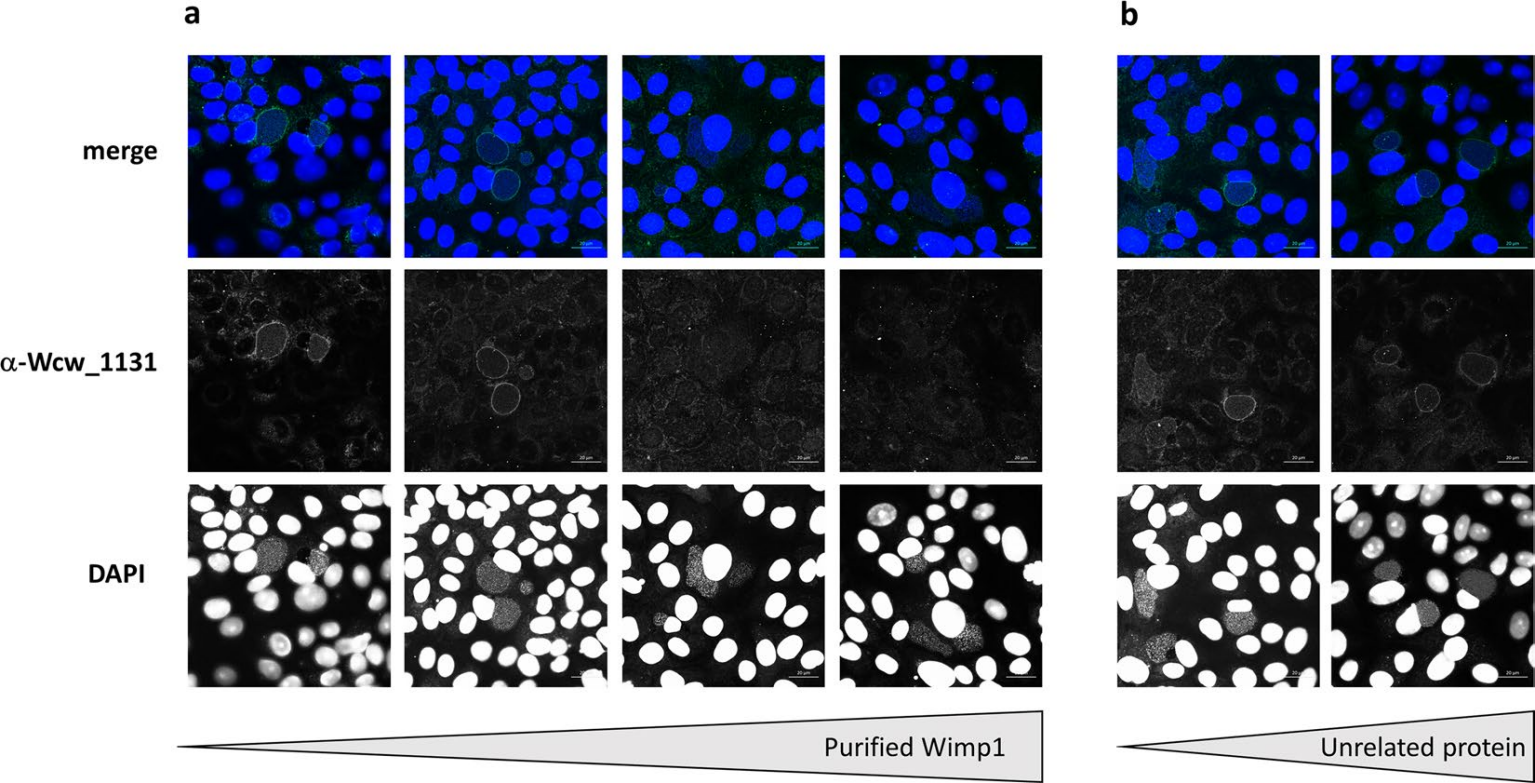
Anti-Wcw_1131 antibody is specific to Wcw_1131. Vero cells infected with *W. chondrophila* during 24 h post-infection were stained with anti Wcw_1131 antibody (green) previously pre-incubated with increasing amounts of **(a)** purified His-Wcw_1131 protein (respectively from left to right no antigen, 0.001 mg/ml, 0.01 mg/ml and 0.05 mg/ml) or **(b)** purified His_Wcw_1618, an unrelated protein (respectively from left to right 0.01 mg/ml and 0.05 mg/ml). Samples were also stained with DAPI (blue) to visualize bacterial DNA. Bar: 20 μm.

Confocal images of Vero cells infected with *W. chondrophila* and fixed at different time points during the replication cycle are presented in Fig. 4a. Protein Wcw_1131 was stained in green with a specific mouse antibody and nucleic acids in blue with DAPI. At 0 h pi, bacteria could be detected by DAPI staining (white arrows) but not with the anti-Wcw_1131 antibody. At 8 and 16 hours pi, the signal corresponding to Wcw_1131 co-localized with bacteria in growing inclusions (white arrows). At 24 hours pi, the green Wcw_1131 signal was clearly located in the membrane of the bacteria-containing inclusions but it was still weakly co-localizing with RBs. At 48 hours, a pronounced staining of the inclusion membranes could be observed. In addition, the green signal was not associated with bacteria anymore, indicating efficient secretion of Wcw_1131. Contrarily to the well-described *C.trachomatis* homotypic inclusion fusion^59^, multiple *W. chondrophila* inclusions do not systematically fuse together and several of them could be observed in one single host cell. No green signal could be detected in non-infected cells or when using the corresponding pre-immune serum (data not shown). However, the signal localized at the inclusion membrane could be due to cross-reactions of the antibody with eukaryotic proteins that decorate the inclusion. To clarify this point, we infected Vero cells with *Estrella lausannensis*, a distantly related bacterium of the *Criblamydiaceae* family and stained the infected cells with the anti-Wcw_1131 antibody. As expected, no signal could be detected on the membrane of *E. lausannensis*-containing inclusions (Fig. 4b). In addition, we performed competition experiments by pre-incubating the anti-Wcw_1131 antibody with increasing amounts of purified Wcw_1131 or of an irrelevant protein before the immunofluorescence staining. Results presented in Fig. 5a clearly demonstrated that the Wcw_1131 signal is abolished following pre-incubation of the antibody with a sufficient amount of purified Wcw_1131, while this is not the case when an unrelated protein is used in the competition assay (Fig. 5b). These immunofluorescence data confirm that the green signal detected at the inclusion membrane is specific for Wcw_1131. This protein is thus the first described inclusion membrane protein of *W. chondrophila*. It was named Wimp1 for Waddlia Inclusion Membrane Protein 1.

## Discussion

In this study, we have used an original approach to identify new virulence proteins of the emerging pathogen *W. chondrophila*. We screened a *W. chondrophila* genomic library using the ability of *E. coli* expressing *W. chondrophila* proteins to resist predation by amoebae. Several studies have demonstrated that the mechanisms involved in resistance to predation by environmental phagocytes are also involved in resistance to phagocytic immune cells and contribute to virulence in human or animal infections^40,60^. Resistance to grazing by protists has thus been widely used to identify virulence factors especially for extracellular bacteria^41–43,61^. However, to our knowledge, only a few studies report the use of *E.coli* transduced with a cosmid library as prey for amoebae^44,62^. Our partial screening of a *W. chondrophila* 1600 clones library resulted in the identification of 28 putative virulence proteins. Among them was a recognized virulence protein, the Macrophage Infectivity Potentiator (Mip)^63^. In the present study, we described one of these newly identified virulence proteins, hypothetical protein Wcw_1131, as the first protein demonstrated to localize to the inclusion membrane of *W. chondrophila*. We named it Wimp1 for Waddlia Inclusion Membrane Protein 1. Given the genetic intractability of *W. chondrophila* we have used a heterologous secretion assay to show that Wimp1 is secreted through *Y. enterocolita* T3SS, indicating that it may be comparably secreted through *W. chondrophila* T3SS. It is surprising that our screening approach using an *E. coli* strain that does not express a T3SS led to the identification of a T3SS effector. It may be serendipitous, for instance, due to the location of another virulence factor on the same cosmid, but it is also possible that Wimp1 is acting as a virulence factor in the context of amoebae feeding on *E. coli* expressing this protein even in absence of a T3SS. Indeed, the *Y. enterocolitica* secretion assays indicated that a small proportion of Wcw_1131 was secreted even when the T3SS was absent (Fig. 2c) or blocked (Supplementary Fig. S2). It is thus possible that this small proportion of Wcw_1131 is secreted by another secretion system and is sufficient to interfere with predation by *A. castellanii*.

Proteins secreted by intracellular chlamydiae to their inclusion membrane are often considered to be virulence factors. Located at the interface between bacteria and host cell, they are very likely to play important roles in the interactions between the pathogen and its host and to allow the chlamydiae to subvert host cell pathways for their own successful replication. Incs of *Chlamydiae* share little sequence similarities between each other but are characterized by a hydrophobic bilobed domain^23,25^. Wimp1 does not display this typical domain, however it may possess other amphipathic characteristics or associate with a yet unknown protein allowing its localization in the *W. chondrophila* inclusion membrane where it can interact with host cell components.

*In silico* predictions based on the typical bilobed hydrophobicity pattern of Incs identified up to hundred putative Incs in chlamydiae but a core set of only 23 of them are shared between 5 different species belonging to the *Chlamydiaceae* family^24^. Thus, despite the large number of host-pathogen interaction mechanisms expected to be shared by all chlamydiae, there is little conservation of Incs between species. This suggests that these bacteria have acquired different sets of eukaryotic-like proteins by horizontal gene transfer from their hosts and that Inc proteins have evolved unique roles related to the bacteria host range. Only 6 of the 23 core Incs have homologs in the genome of *W. chondrophila*, a feature that might reflect the large differences observed in host range and survival mechanisms between *Chlamydiaceae* and *Chlamydia*-related bacteria^2,64^.

Inc proteins extensively modify the chlamydial inclusion membrane and they sometimes represent up to 10% of the coding capacity of the bacterium^24^. Surprisingly, even if *Chlamydia*-related bacteria have much larger genomes than *Chlamydiaceae*, only a small proportion of their proteins exhibit the typical Inc bilobed hydrophobic domain. Indeed, only 23 proteins of *Protochlamydia amoeobophila* display this hydrophobicity pattern^65^. However, it is very likely that multiple proteins also decorate the inclusion membrane of *Chlamydia*-related bacteria, particularly considering their broad host range and ability to resist digestion by amoebae. Since a significant number of known Inc proteins of *C. trachomatis* exhibit some sequence similarities with specific eukaryotic domains, we have searched the complete *W. chondrophila* ORFeome for protein sequences that are specific to this bacterial species and have a best blast hit against eukaryotic proteins. This analysis allowed the identification of 21 putative inclusion membrane proteins specific to *W. chondrophila*. These proteins are considered to be first secreted by the T3SS and then subsequently relocated in the inclusion membrane facing the host cell cytoplasm. We demonstrated in the present study that Wcw_1131 is indeed secreted by T3SS and located in the inclusion membrane from 24 hours post infection. Five of the 21 *W. chondrophila*-specific proteins exhibiting a BBH against eukaryotic proteins have a predicted N-terminal signal peptide allowing translocation across the inner membrane by the Sec machinery. The presence of this signal peptide probably prevents secretion by the T3SS. Among the 16 remaining proteins, four, including Wcw_1131, contain a Ras GEF or a Rho GEF domain. These domains are thought to regulate Ras GTPases (or Rho GTPases) signal transduction. They have numerous effects on cellular differentiation and proliferation, cytoskeleton rearrangements, vesicular trafficking and nuclear transport^66^. Interestingly, Ras GEF proteins are found only in *Legionella, Piscirickettsia, Coxiella and Berkiella spp*. as well as in three *Chlamydiae* families: *Parachlamydiaceae, Criblamydiaceae* and *Waddliaceae*. All these bacterial species are amoebae-resisting organisms. In addition, all the amoebal genomes included in RefSeq (n = 10) encode protein(s) with Ras GEF domains. It is thus probable that genetic exchanges among intra-amoebal microorganisms or between microorganisms and their amoebal hosts resulted in the acquisition of these Ras GEF proteins, since amoebae are melting pots for gene exchanges^2,67,68^.

The transcription profile of gene *wcw_1131* revealed its early expression during the replication cycle suggesting that the corresponding protein could interfere with early mechanisms of host cell response to infection. Wcw_1131 could play a role in the intra-amoebal survival of *W. chondrophila*, an ability probably related to the rapid escape of the bacterium from the endocytic pathway and its replication in vacuoles in close association with endoplasmic reticulum markers, such as calnexin^14^. Similarly, intracellular survival of *Legionella pneumophila* depends on the secretion of effectors by the Type 4 Secretion System that avoid phagosome-endosome fusion and help *Legionella* to relocate with the endoplasmic reticulum. Some of these *Legionella* effectors have similar functions in both amoebae and macrophages^69,70^. Alternatively, Wcw_1131 could be implicated in the recruitment of mitochondria around the *W. chondrophila* inclusion, an event that occurs very rapidly after bacterial entry in the host^14^.

To conclude, this work reports the results of a screen for virulence proteins, using a *W. chondrophila* cosmid library in *E. coli* in an assay monitoring resistance of these transduced bacteria to predation by amoebae. This screening led to the identification of 23 cosmids harboring multiple genes and conferring resistance to *E. coli*. Candidate virulence proteins present on these cosmids were further identified by bioinformatics analyses. So far, only one of them was studied in detail, but this is a promising result, as we identified the first *W. chondrophila* inclusion membrane protein. This species-specific virulence protein is secreted by the T3SS and located in the inclusion membrane. Its temporal gene and protein expression indicate that it is produced at the beginning of the replication cycle, probably in order to rapidly interfere with cellular processes and to establish favorable conditions for *W. chondrophila* survival and replication. Further experiments are now needed to define its precise role in the host-pathogen interactions.

## Methods

### Cell culture and bacterial strains

Vero cells (ATCC CCL-81) were routinely maintained at 37 °C, 5% CO_2_ in high glucose Dulbecco’s modified minimal essential medium (DMEM, PAN Biotech, Aidenbach, Germany) supplemented with 10% foetal calf serum (Gibco, Thermo Fisher Scientific, Waltham, USA). *Waddlia chondrophila* strain ATCC VR-1470 was grown at 32 °C within *Acanthamoeba castellanii* strain ATCC 30010 as described elsewhere^71^. For infection of Vero cells, bacteria were recovered from a 4 day-old amoebal co-culture and filtered through a 5-μm filter (Millipore, Carrigtwohill, Ireland) to eliminate trophozoites and cysts.

### Cosmid library and predation assay using amoebae

A *W. chondrophila* WSU86–1044 cosmid library containing about 1600 clones and covering 53% of the genome was obtained in *E. coli* EPI100-T1^R^ by Amplicon Express (Pullman, USA). Briefly, *W. chondrophila* genomic DNA fragments of 20 to 40 kb (average size 30 kb) were inserted in pWEB^TM^ vectors and packed in bacteriophages, which were then used to transduce *E. coli*. Cosmids were partially sequenced by Amplicon Express using T7 and M13 universal primers. They were not evenly distributed along the genome but rather clustered in some hot spots regions.

The amoebal predation assay was performed on *Acanthamoeba castellanii* as described in Froquet *et al*.^61^. Briefly, overnight cosmid-containing *E. coli* cultures were diluted at 10^8^ bacteria /ml and 500 μl were spread on SM agar plates (1% glucose, 1% proteose peptone, 0.1% yeast extract, 4 mM MgSO_4_, 14 mM KH_2_PO_4_, 3.4 mM K_2_HPO_4_, 2% bacto agar). *Acanthamoeba castellanii* strain ATCC 30010 were grown at 25 °C in PYG broth and resuspended in PAS buffer as described in Jacquier *et al*.^71^. 10^6^, 10^5^, 10^4^ and 10^3^ amoebae were spotted on the lawn of *E. coli* and plates were incubated for 3 days at 28 °C in a humidified atmosphere. Pictures were taken with AlphaImager 3400 ^TM^ (Alpha-InnoTec, St_Sulpice, Switzerland) and ImageJ software was used to measure diameters of lysis plaques.

Cosmids of interest were sequenced and mapped with Geneious software against the *W. chondrophila* genome with the following parameters: High/medium sensitivity and up to 5 times iteration^19^.

### Bioinformatic analysis

Complete genome and proteome sequences of *W. chondrophila* WSU 86–1044 have been downloaded from chlamdb database (https://chlamdb.ch)^72^. Sequences were submitted to 3 sequence-based prediction programs to predict potential protein secretion through T3SS. Effective T3 (https://effectors.csb.univie.ac.at)^49^, BPBAac (https://biocomputer.bio.cuhk.edu.hk/T3DB/BPBAac.php)^51^ and Modlab^50^ are online prediction tools based on an naïve Bayes algorithms, and trained on large dataset to recognize specific peptides in N-terminal protein region of secreted T3SS effectors.

The identification of homologs of *W. chondrophila* proteins, the identification of *W. chondrophila-*specific proteins and homology search against the RefSeq database to identify best hits with eukaryotic sequences were based on data and methods published in Pillonel *et al*.^73^.

### *Yersinia enterocolitica* Type 3 secretion assays

T3S assays using *Y. enterocolitica* ΔHOPEMT and ΔHOPEMT ΔYscU strains were performed as described in da Cunha *et al*.^53^. Briefly, Wcw_1131 full gene was cloned in pLJM3 with a C-terminal V5 epitope tag and transformed in the T3-proficient (ΔHOPEMT) and T3-deficient (ΔHOPEMT ΔYscU) strains. Bacteria were grown for 2 hours at 27 °C in BHI medium supplemented with 20 mM sodium oxalate, 0.4% glucose, and 20 mM MgCl_2_. Activation of the *yop* regulon was obtained by temperature shift to 37 °C in a water bath. After 4 hours incubation, absorbance of the culture was measured at OD_600_ and bacteria were pelleted by 1 minute centrifugation at 14′000 rpm. Pellets were resuspended in SDS loading buffer and supernatants were precipitated with trichloroacetic acid before resuspension in SDS loading buffer. Both fractions were normalized according to the OD _600_ values and analyzed by immunoblot. Signal intensities were measured with Image J and the percentage of secretion was calculated as the ratio between the amount of secreted protein relative to the total amount of protein.

Type 3-dependent secretion of Wcw_1131 was also assessed using a T3S assay described in Sorg *et al*.^74^. Briefly, Wcw_1131 full gene was cloned in pBAD-DEST49 Gateway destination vector (Invitrogen, Thermo Fisher Scientific, Waltham, USA), and transformed in *Y. enterocolitica* MRS40 (pNG40031) strain^75^. Bacteria were grown in BHI supplemented with glycerol (4 mg/mL) and 20 mM MgCl_2_. Expression of Wcw_1131 was induced by adding 0.2% L-arabinose, and activation of the *yop* regulon was obtained by temperature shifting from 28 °C to 37 °C. Permissive secretion through T3SS conditions were obtained by chelating calcium with 20 mM sodium oxalate. After 4 hours, samples were normalized based on absorbance at OD_600_, and bacteria were pelleted by centrifugation (17000 g, 10 minutes, 4 °C). Pellets were resuspended in SDS loading buffer and supernatants were precipitated with trichloroacetic acid before resuspension in SDS loading buffer. Both fractions were analyzed by immunoblot. Signal intensities were measured with Image J and the ratio of values detected in supernatant versus pellet fractions were calculated for each tested condition.

### Statistical analyses

Statistical analyses were performed using GraphPad Prism 8.01 software (GraphPad Software Inc., La Jolla CA, USA). Percentage of secretion calculated in *Y. enterocolitica* Type 3 secretion assays were compared using a non-parametric Mann-Whitney test with 95% confidence interval and two-tailed P value. ** indicates p value < 0.01.

### Purification of recombinant Wcw_1131 and antibody production

Wcw_1131 full gene was cloned using NdeI and SacI restriction sites in pCWR547, a plasmid allowing addition of a N-terminal 6His tag to the protein^76^. The 6His tag is followed by a recognition site for the SUMO protease. Recombinant protein was expressed in BL21 *E.coli* and purified on a Ni-NTA agarose (Quiagen, Hombrechtikon, Switzerland) under denaturing conditions following the manufacturer’s protocol. Removal of the 6His tag was performed with SUMO protease (Invitrogen, Thermo Fisher Scientific, Waltham, USA) according to supplier’s protocol. Polyclonal mouse and rabbit antibodies were obtained from Eurogentec (Liège, Belgium).

### Infection procedure

Epithelial cells were seeded, 1 day before infection, at 2.5 × 10^5^ cells per well in 24-wells microplates for immunofluorescence or at 4 × 10^6^ cells per 25 cm^2^ flasks for RNA extraction and total protein preparation. Cells were infected as described in Kebbi Beghdadi *et al*.^34^.with a 1/2000 dilution of *W. chondrophila* grown in *A. castellanii*, which corresponds to an MOI of 2–3, as estimated with a *W. chondrophila* specific real time quantitative PCR^77^. Alternatively, Vero cells were infected with a 1/2000 dilution of *E. lausannensis* grown in *A. castellanii* (MOI 2–3)^34^.

### Gene expression

At different time points after infection, culture supernatant and Vero cells harvested by scraping were collected in TRIzol (AmbionR, Life Technologies, Thermo Fisher Scientific, Waltham, USA) and RNA was extracted as described in Chomczynski and Mackey^78^. cDNA was produced using random primers and the GoScript Reverse Transcription kit (Promega, Dübendorf, Switzerland). Quantitative PCR was performed on total cDNA using I Taq SYBRGreen technology (BioRad, Cressier, Switzerland), 4 μl of 1/25 cDNA sample and 300 nM (16S rRNA) or 200 nM (wcw_1131) of the following primers:

16S rRNA for: 5′ GGCCCTTGGGTCGTAAAGTTCT 3′

16 S rRNA rev: 5′ CGGAGTTAGCCGGTGCTTCT 3′

wcw_1131 for: 5′ CCGCCTCATGTACAACCCTT 3′

wcw_1131 rev 5′: CCTGAAGGGTCGATGCAGTT 3′

Cycling conditions were 10 min at 95 °C, followed by 40 cycles of 15 s at 95 °C and 1 min at 60 °C. Amplification and detection of PCR products were performed with the StepOne Real-Time PCR System (Applied Biosystems, Zug, Switzerland). qRT-PCR results were analyzed using 16 S rRNA gene as the endogenous control and 48 h pi as the reference time point.

### Immunoblots

#### Secretion assays

Pellet and precipitated supernatant fractions were loaded on SDS gels and proteins were separated by electrophoresis. Immunoblots were performed as described in Kebbi Beghdadi *et al*.^58^. Mouse monoclonal anti-V5 epitope antibody (Invitrogen, Thermo Fisher Scientific, Waltham, USA) was used at 1/5000 dilution and incubated for 2 hours at room temperature or overnight at 4 °C. Rabbit polyclonal anti-MreB was used at 1/5000 dilution and incubated overnight at 4 °C. Secondary antibodies, goat anti-mouse IgG-HRP and donkey anti-rabbit IgG-HRP (BioRad, Cressier, Switzerland) were used at 1/3000 dilution and incubated for 1 hour at room temperature. Immunoblots were revealed with ECL^TM^ Prime Western Blotting Detection Reagent (Amersham, GE Healthcare, Glattbrugg, Switzerland) and analysed on a ImageQuant LAS4000 mini (Amersham, GE Healthcare, Glattbrugg, Switzerland). Image J software was used to quantify signal intensity.

#### *W. chondrophila infected cells*

At different time points after infection, culture supernatant and infected Vero cells harvested by scraping were centrifuged 5 minutes at 12′000 g. 50 μl were kept for DNA extraction with Wizard SV genomic DNA extraction kit (Promega, Dübendorf, Switzerland) and quantification using *W. chondrophila* specific qPCR^77^ as described in Kebbi Beghdadi *et al*.^34^. The pellet corresponding to one T25 flask was washed once with PBS and resuspended in 500 μl of SDS PAGE loading buffer. Proteins were separated by SDS PAGE and transferred on a nitrocellulose membrane. Immunoblots were performed as described in Kebbi Beghdadi *et al*.^58^. Rabbit anti-Wcw_1131 antibody was used at a 1/1000 dilution and incubated overnight at 4 C. Secondary goat anti-rabbit-HRP conjugated antibody (Promega, Dübendorf, Switzerland) was used at 1/3000 dilution and incubated for 1 hour at room temperature. Immunoblots were revealed with ECL^TM^ Prime Western Blotting Detection Reagent (Amersham, GE Healthcare, Glattbrugg, Switzerland) and analysed on a ImageQuant LAS4000 mini (Amersham, GE Healthcare, Glattbrugg, Switzerland). The signal intensities were measured with ImageJ, normalized according to the number of bacteria present in each sample (determined by quantitative PCR) and expressed as a percentage of the maximum value.

### Immunofluorescence and confocal microscopy

At 0, 8, 16, 24 and 48 hours post infection, infected Vero cells grown on glass coverslips were fixed with ice-cold methanol for 5 min, washed three times with PBS and incubated in blocking solution (PBS, 0.01% NaN_3_, 1% BSA) at 4 °C. Coverslips were incubated for 2 h at room temperature with a mouse anti-Wcw_1131 antibody diluted 1/200 in PBS, 0.1% saponin, 1% BSA. For protein-antibody competition experiments, the anti-Wcw_1131 antibody (final dilution 1/200) was pre-incubated during 90 minutes at room temperature with purified proteins His-Wcw_1131 or His-Wcw_1618 at final concentrations of 0.05, 0.01 or 0.001 mg/ml. After three washing steps in PBS, 0.1% saponin, coverslips were incubated for 1 h at room temperature with a 1/500 dilution of AlexaFluor 488-conjugated goat anti-mouse (Life Technologies, Thermo Fisher Scientific, Waltham, USA) and a 1/30000 dilution of DAPI dilactate (4′,6-Diamidino-2-Phenylindole Dihydrochloride, Molecular Probes, Thermo Fisher Scientific, Waltham, USA). After washing twice with PBS 0.1% saponin, once with PBS and once with deionized water, the coverslips were mounted onto glass slides using Mowiol (Sigma-Aldrich, Buchs, Switzerland). Cells were observed under a confocal microscope (Zeiss LSM 710 or 780 Quasar Confocal Microscope, Feldbach, Switzerland).

## Supplementary information

Supplementary tables and figures

## Data Availability

The datasets generated and/or analyzed during the current study are available from the corresponding author on reasonable request.

## References

[1] Pillonel, T., Bertelli, C. & Greub, G. Environmental Metagenomic Assemblies Reveal Seven New Highly Divergent Chlamydial Lineages and Hallmarks of a Conserved Intracellular Lifestyle. *Front Microbiol* **9**, 10.3389/fmicb.2018.00079 (2018). 10.3389/fmicb.2018.00079 PMC5826181 29515524

[2] Greub, G. The medical importance of *Chlamydiae*. *Clin Microbiol Infect* **15**, 2-3, CLM2632 (2009). 10.1111/j.1469-0691.2008.02632.x 19220333

[3] Taylor-Brown A, Vaughan L, Greub G, Timms P, Polkinghorne A (2015). Twenty years of research into *Chlamydia*-like organisms: a revolution in our understanding of the biology and pathogenicity of members of the phylum *Chlamydiae*. Pathogens and disease.

[4] Henning K (2002). Neospora caninum and Waddlia chondrophila strain 2032/99 in a septic stillborn calf. Veterinary Microbiology.

[5] Rurangirwa FR, Dilbeck PM, Crawford TB, McGuire TC, McElwain TF (1999). Analysis of the 16S rRNA gene of micro-organism WSU 86-1044 from an aborted bovine foetus reveals that it is a member of the order *Chlamydiales*: proposal of *Waddliaceae* fam. nov., *Waddlia chondrophila* gen. nov., sp. nov. International journal of systematic bacteriology.

[6] Barkallah M (2014). Survey of infectious etiologies of bovine abortion during mid- to late gestation in dairy herds. PLoS One.

[7] Blumer S (2011). *Waddlia, Parachlamydia* and *Chlamydiaceae* in bovine abortion. Vet Microbiol.

[8] Baud D (2011). *Waddlia chondrophila*: From Bovine Abortion to Human Miscarriage. Clinical infectious diseases.

[9] Baud D (2014). Role of *Waddlia chondrophila* placental infection in miscarriage. Emerg Infect Dis.

[10] Baud D, Greub G (2011). Intracellular bacteria and adverse pregnancy outcomes. Clin Microbiol Infect.

[11] Haider, S., Collingro, A., Walochnik, J., Wagner, M. & Horn, M. *Chlamydia*-like bacteria in respiratory samples of community-acquired pneumonia patients. *FEMS Microbiol Lett* **281**, 198-202, FML1099 (2008). 10.1111/j.1574-6968.2008.01099.x 18312573

[12] Hornung S (2015). Role of *Chlamydia trachomatis* and emerging *Chlamydia*-related bacteria in ectopic pregnancy in Vietnam. Epidemiology and infection.

[13] Verweij SP (2015). *Waddlia chondrophila* and *Chlamydia trachomatis* antibodies in screening infertile women for tubal pathology. Microbes Infect.

[14] Croxatto, A. & Greub, G. Early intracellular trafficking of *Waddlia chondrophila* in human macrophages. *Microbiology* **156**, 340-355, mic.0.034546-0 (2010). 10.1099/mic.0.034546-0 19926655

[15] Kebbi-Beghdadi C, Cisse O, Greub G (2011). Permissivity of Vero cells, human pneumocytes and human endometrial cells to *Waddlia chondrophila*. Microbes and infection.

[16] Elwell C, Mirrashidi K, Engel J (2016). *Chlamydia* cell biology and pathogenesis. Nat Rev Microbiol.

[17] Beeckman, D. S. & Vanrompay, D. C. Bacterial secretion systems with an emphasis on the chlamydial Type III secretion system. *Curr Issues Mol Biol* **12**, 17-41, v12/17 (2010). 19605938

[18] Peters J, Wilson DP, Myers G, Timms P, Bavoil PM (2007). Type III secretion a la Chlamydia. Trends Microbiol.

[19] Bertelli C (2010). The *Waddlia* genome: a window into chlamydial biology. PLoS One.

[20] Collingro, A. *et al*. Unity in Variety - the Pan-Genome of the *Chlamydiae*. *Mol Biol Evol*, msr161 (2011). 10.1093/molbev/msr161 PMC3247790 21690563

[21] Li Z (2008). Characterization of fifty putative inclusion membrane proteins encoded in the *Chlamydia trachomatis* genome. Infect Immun.

[22] Weber MM, Bauler LD, Lam J, Hackstadt T (2015). Expression and localization of predicted inclusion membrane proteins in *Chlamydia trachomatis*. Infect Immun.

[23] Bannantine JP, Griffiths RS, Viratyosin W, Brown WJ, Rockey DD (2000). A secondary structure motif predictive of protein localization to the chlamydial inclusion membrane. Cell Microbiol.

[24] Lutter EI, Martens C, Hackstadt T (2012). Evolution and conservation of predicted inclusion membrane proteins in chlamydiae. Comp Funct Genomics.

[25] Rockey DD, Scidmore MA, Bannantine JP, Brown WJ (2002). Proteins in the chlamydial inclusion membrane. Microbes Infect.

[26] Alzhanov DT, Weeks SK, Burnett JR, Rockey DD (2009). Cytokinesis is blocked in mammalian cells transfected with *Chlamydia trachomatis* gene CT223. BMC Microbiol.

[27] Cortes, C., Rzomp, K. A., Tvinnereim, A., Scidmore, M. A. & Wizel, B. *Chlamydia pneumoniae* inclusion membrane protein Cpn0585 interacts with multiple Rab GTPases. *Infect Immun* **75**, 5586-5596, IAI.01020-07 (2007). 10.1128/IAI.01020-07 PMC2168330 17908815

[28] Delevoye C (2008). SNARE protein mimicry by an intracellular bacterium. PLoS Pathog.

[29] Lutter EI, Barger AC, Nair V, Hackstadt T (2013). *Chlamydia trachomatis* inclusion membrane protein CT228 recruits elements of the myosin phosphatase pathway to regulate release mechanisms. Cell Rep.

[30] Almeida F, Luis MP, Pereira IS, Pais SV, Mota LJ (2018). The Human Centrosomal Protein CCDC146 Binds *Chlamydia trachomatis* Inclusion Membrane Protein CT288 and Is Recruited to the Periphery of the Chlamydia-Containing Vacuole. Frontiers in cellular and infection microbiology.

[31] Hackstadt T, Scidmore-Carlson MA, Shaw EI, Fischer ER (1999). The *Chlamydia trachomatis* IncA protein is required for homotypic vesicle fusion. Cell Microbiol.

[32] Weber MM (2017). Absence of Specific *Chlamydia trachomatis* Inclusion Membrane Proteins Triggers Premature Inclusion Membrane Lysis and Host Cell Death. Cell Rep.

[33] Kebbi-Beghdadi C, Batista C, Greub G (2011). Permissivity of fish cell lines to three Chlamydia-related bacteria: *Waddlia chondrophila, Estrella lausannensis* and *Parachlamydia acanthamoebae*. FEMS Immunol Med Microbiol.

[34] Kebbi-Beghdadi C, Fatton M, Greub G (2015). Permissivity of insect cells to *Waddlia chondrophila*, *Estrella lausannensis* and *Parachlamydia acanthamoebae*. Microbes Infect.

[35] Coulon C (2012). Amoebal host range, host-free survival and disinfection susceptibility of environmental *Chlamydiae* as compared to *Chlamydia trachomatis*. FEMS Immunol Med Microbiol.

[36] Elwell, C. & Engel, J. N. Drosophila melanogaster S2 cells: a model system to study *Chlamydia* interaction with host cells. *Cell Microbiol* **7**, 725-739, CMI508 (2005). 10.1111/j.1462-5822.2005.00508.x PMC1236988 15839901

[37] Heuer D (2009). *Chlamydia* causes fragmentation of the Golgi compartment to ensure reproduction. Nature.

[38] Hackstadt T, Scidmore MA, Rockey DD (1995). Lipid metabolism in *Chlamydia trachomatis*-infected cells: directed trafficking of Golgi-derived sphingolipids to the chlamydial inclusion. Proceedings of the National Academy of Sciences of the United States of America.

[39] Tosetti N, Croxatto A, Greub G (2014). Amoebae as a tool to isolate new bacterial species, to discover new virulence factors and to study the host-pathogen interactions. Microb Pathog.

[40] Sun S, Noorian P, McDougald D (2018). Dual Role of Mechanisms Involved in Resistance to Predation by Protozoa and Virulence to Humans. Front Microbiol.

[41] Adiba S, Nizak C, van Baalen M, Denamur E, Depaulis F (2010). From grazing resistance to pathogenesis: the coincidental evolution of virulence factors. PLoS One.

[42] Alibaud L (2008). *Pseudomonas aeruginosa* virulence genes identified in a *Dictyostelium* host model. Cell Microbiol.

[43] Cosson P (2002). *Pseudomonas aeruginosa* virulence analyzed in a *Dictyostelium discoideum* host system. J Bacteriol.

[44] Waterfield NR (2008). Rapid Virulence Annotation (RVA): identification of virulence factors using a bacterial genome library and multiple invertebrate hosts. Proc Natl Acad Sci USA.

[45] Quilliam LA, Khosravi-Far R, Huff SY, Der CJ (1995). Guanine nucleotide exchange factors: activators of the Ras superfamily of proteins. Bioessays.

[46] Boquet P (2000). Small GTP binding proteins and bacterial virulence. Microbes Infect.

[47] Makrinos DL, Bowden TJ (2017). Growth characteristics of the intracellular pathogen, *Piscirickettsia salmonis*, in tissue culture and cell-free media. J Fish Dis.

[48] Mehari YT (2016). Description of ‘Candidatus Berkiella aquae’ and ‘Candidatus Berkiella cookevillensis’, two intranuclear bacteria of freshwater amoebae. Int J Syst Evol Microbiol.

[49] Arnold R (2009). Sequence-based prediction of type III secreted proteins. PLoS Pathog.

[50] Lower M, Schneider G (2009). Prediction of type III secretion signals in genomes of gram-negative bacteria. PLoS One.

[51] Wang Y, Huang H, Sun M, Zhang Q, Guo D (2012). T3DB: an integrated database for bacterial type III secretion system. BMC Bioinformatics.

[52] Bauler LD, Hackstadt T (2014). Expression and targeting of secreted proteins from *Chlamydia trachomatis*. J Bacteriol.

[53] da Cunha M (2014). Identification of type III secretion substrates of *Chlamydia trachomati*s using *Yersinia enterocolitica* as a heterologous system. BMC Microbiol.

[54] Pais SV, Milho C, Almeida F, Mota LJ (2013). Identification of novel type III secretion chaperone-substrate complexes of *Chlamydia trachomatis*. PLoS One.

[55] Agrain C, Sorg I, Paroz C, Cornelis GR (2005). Secretion of YscP from *Yersinia enterocolitica* is essential to control the length of the injectisome needle but not to change the type III secretion substrate specificity. Mol Microbiol.

[56] Keyser P, Elofsson M, Rosell S, Wolf-Watz H (2008). Virulence blockers as alternatives to antibiotics: type III secretion inhibitors against Gram-negative bacteria. J Intern Med.

[57] Chen YS (2014). The *Chlamydia trachomatis* type III secretion chaperone Slc1 engages multiple early effectors, including TepP, a tyrosine-phosphorylated protein required for the recruitment of CrkI-II to nascent inclusions and innate immune signaling. PLoS Pathog.

[58] Kebbi-Beghdadi, C. *et al*. OmpA family proteins and Pmp-like autotransporter: new adhesins of *Waddlia chondrophila*. *Pathogens and disease* **73**, 10.1093/femspd/ftv035 (2015). 10.1093/femspd/ftv035 25986220

[59] Van Ooij C, Homola E, Kincaid E, Engel J (1998). Fusion of *Chlamydia trachomatis*-containing inclusions is inhibited at low temperatures and requires bacterial protein synthesis. Infect Immun.

[60] Greub G, Raoult D (2004). Microorganisms resistant to free-living amoebae. Clin Microbiol Rev.

[61] Froquet R, Lelong E, Marchetti A, Cosson P (2009). *Dictyostelium discoideum*: a model host to measure bacterial virulence. Nat Protoc.

[62] Dorati Federico, Barrett Glyn, Sanchez-Contreras Maria, Arseneault Tanya, José Mateo, Studholme David, Murillo Jesús, Caballero Primitivo, Waterfield Nicholas, Arnold Dawn, Shaw Liz, Jackson Robert (2018). Coping with Environmental Eukaryotes; Identification of Pseudomonas syringae Genes during the Interaction with Alternative Hosts or Predators. Microorganisms.

[63] Herrmann Michael, Schuhmacher Alexander, Mühldorfer Inge, Melchers Klaus, Prothmann Christian, Dammeier Sascha (2006). Identification and characterization of secreted effector proteins of Chlamydophila pneumoniae TW183. Research in Microbiology.

[64] Horn M (2008). *Chlamydiae* as symbionts in eukaryotes. Annual review of microbiology.

[65] Heinz E (2010). Inclusion membrane proteins of *Protochlamydia amoebophila* UWE25 reveal a conserved mechanism for host cell interaction among the Chlamydiae. J Bacteriol.

[66] Simanshu DK, Nissley DV, McCormick F (2017). RAS Proteins and Their Regulators in Human Disease. Cell.

[67] Bertelli C, Greub G (2012). Lateral gene exchanges shape the genomes of amoeba-resisting microorganisms. Frontiers in cellular and infection microbiology.

[68] Moliner C, Fournier PE, Raoult D (2010). Genome analysis of microorganisms living in amoebae reveals a melting pot of evolution. FEMS microbiology reviews.

[69] Finsel I, Hilbi H (2015). Formation of a pathogen vacuole according to *Legionella pneumophila*: how to kill one bird with many stones. Cell Microbiol.

[70] So EC, Mattheis C, Tate EW, Frankel G, Schroeder GN (2015). Creating a customized intracellular niche: subversion of host cell signaling by *Legionella* type IV secretion system effectors. Can J Microbiol.

[71] Jacquier, N., Aeby, S., Lienard, J. & Greub, G. Discovery of new intracellular pathogens by amoebal coculture and amoebal enrichment approaches. *Journal of visualized experiments: JoVE*, e51055, 10.3791/51055 (2013). 10.3791/51055 PMC3964206 24192667

[72] Pillonel, T., Tagini, F., Bertelli, C. & Greub, G. ChlamDB: a comparative genomics database of the phylum Chlamydiae and other members of the Planctomycetes-Verrucomicrobiae-Chlamydiae superphylum. *Nucleic Acids Res*, 10.1093/nar/gkz924 (2019). 10.1093/nar/gkz924 PMC7145651 31665454

[73] Pillonel T (2019). Sequencing the Obligate Intracellular Rhabdochlamydia helvetica within Its Tick Host Ixodes ricinus to Investigate Their Symbiotic Relationship. Genome biology and evolution.

[74] Sorg I (2007). YscU recognizes translocators as export substrates of the *Yersinia* injectisome. Embo J.

[75] Cornelis G, Vanootegem JC, Sluiters C (1987). Transcription of the yop regulon from *Y. enterocolitica* requires trans acting pYV and chromosomal genes. Microb Pathog.

[76] Radhakrishnan SK, Pritchard S, Viollier PH (2010). Coupling prokaryotic cell fate and division control with a bifunctional and oscillating oxidoreductase homolog. Dev Cell.

[77] Goy G, Croxatto A, Posfay-Barbe KM, Gervaix A, Greub G (2009). Development of a real-time PCR for the specific detection of *Waddlia chondrophila* in clinical samples. Eur J Clin Microbiol Infect Dis.

[78] Chomczynski P, Mackey K (1995). Short technical reports. Modification of the TRI reagent procedure for isolation of RNA from polysaccharide- and proteoglycan-rich sources. BioTechniques.

