# Supplementary material for: A predation assay using amoebae to screen for virulence factors unearthed the first W. chondrophila inclusion membrane protein
Source: Sci Rep. 2019 Dec 20;9:19485. doi: 10.1038/s41598-019-55511-1 (PMC6925127; doi:10.1038/s41598-019-55511-1)
Supplement: Supplementary file 1 — Supplementary tables and figures [file 41598_2019_55511_MOESM1_ESM.pdf]

A predation assay using amoebae to screen for virulence factors unearthed the first *W. chondrophila* inclusion membrane protein

C. Kebbi-Beghdadi°, L. Pilloux°, A. Croxatto, N. Tosetti, T. Pillonel and G. Greub*

° Equally contributed

Center for Research on Intracellular Bacteria, Institute of Microbiology, Centre Hospitalier Universitaire Vaudois, Lausanne, Switzerland

*Corresponding author:

Prof. Gilbert Greub

Institute of Microbiology

Rue du Bugnon 48

1011 Lausanne

Switzerland

Tel: 0041 21 314 4979

Fax 0041 21 314 4060

e-mail: gilbert.greub@chuv.ch

Locus tag	Cosmid	Size (bp)	Function	Family	Domain	SignalP/TMHMM
wcw_0023	4B06, 5E07, 5G08	756	Virulence protein	Uncharacterised protein family (UPF0137)		SP
wcw_0028	4B06, 5E07, 5G08	915	Peptidyl-prolyl cis-trans isomerase Mip precursor			SP
wcw_0039	4B06, 5E07, 5G08	618	Peroxiredoxin 1	Alkyl hydroperoxide reductase subunit C/ Thiol specific antioxidant family		
wcw_0460	4A03	414	Hypothetical protein	Thioesterase-like superfamily		
wcw_0463	4A03	1191	Hypothetical protein	Peptidase family M23		SP
wcw_0932	4H10	480	DNA protection during starvation family protein	Ferritin-like superfamily		
wcw_0936	4H10	532	Type IV secretion System effector, Hcp			SP
wcw_0946	4H10	666	Hypothetical protein	CAAX amino terminal protease		TM
wcw_0947	4H10	771	Hypothetical protein		Protein phosphatase 2C	
wcw_0954	4H10	1314	Hypothetical protein		Peptidase M16 domain	SP
wcw_1021	2A10	378	Hypothetical protein	Immunity protein 33		
wcw_1037	2A10	1299	Hypothetical protein		Transporter associated domain	TM
**wcw_1131**	**4CO2, 4H07**	**1941**	**Hypothetical protein**		**Ras GEF domain**	
wcw_1138	4CO2, 4H07	1020	tRNA threonylcarbamoyladenosine modification protein TsaD		Gcp-like domain	
wcw_1143	4CO2, 4H07	1044	Hypothetical protein	Vitamine K epoxide reductase family		SP/TM
wcw_1238	3D10	1632	Hypothetical protein			hydrophobic bi-lobed
wcw_1266	3D10	429	Hypothetical protein	Host attachment protein		
wcw_1268	3D10	1888	Hypothetical protein	Tetratricopeptide-like helical		SP
wcw_1814	several	3318	Serine/threonine protein kinase			TM
wcw_1815	several	3081	Multidrug resistance protein MdtC			SP/TM
wcw_1816	several	1083	Multidrug resistance protein mdtA precursor		RND efflux pump, membrane fusion protein	SP
wcw_1817	several	1452	Porin	Outer membrane efflux protein		SP
wcw_1826	several	1437	RND transporter	Outer membrane efflux protein		SP
wcw_1827	several	3120	Multidrug efflux RND transporter permease subunit		Acriflavin resistance protein	TM
wcw_1828	several	1157	Hemolysin D		RND efflux pump, membrane fusion protein	SP
wcw_1835	several	624	Hydrolase	Haloacid dehalogenase-like hydrolase		
wcw_1836	several	954	NAD-dependent epimerase			SP
wcw_1837	several	1050	Tyrosine-protein phosphatase			TM

Supplementary Table S1: *W. chondrophila* putative virulence proteins located on cosmids identified as virulent in an assay measuring resistance to predation by amoebae.

Locus tag	Hit description	Id (%)	Kingdom	Phylum	Product
wcw_0375	Citrus sinensis (sweet orange)	30.43	Viridiplantae	Streptophyta	Uncharacterized protein LOC102607864 isoform X1
wcw_0387	Brassica napus (rape)	22.87	Viridiplantae	Streptophyta	Protein NETWORKED 4B-like
wcw_0396	*Dictyostelium purpureum*	29.17	-	-	Ras guanine nucleotide exchange factor
wcw_0431	*Bathycoccus prasinos*	30.89	Viridiplantae	Chlorophyta	Predicted protein
wcw_0441	*Acanthamoeba castellanii str. Neff*	21.32	-	-	Hypothetical protein ACA1_321560
wcw_0490	*Apis cerana (Asiatic honeybee)*	26.42	Metazoa	Arthropoda	Rac guanine nucleotide exchange factor JJ-like
wcw_0577	*Danio rerio (zebrafish)*	19.77	Metazoa	Chordata	Nesprin-1 isoform X1
wcw_0594	*Haplochromis burtoni (Burton's mouthbrooder)*	18.74	Metazoa	Chordata	Myosin-7-like isoform X1
wcw_0827	*Aplysia californica (California sea hare)*	26.79	Metazoa	Mollusca	Uncharacterized protein LOC101848656
wcw_0970	*Colobus angolensis palliatus*	20.88	Metazoa	Chordata	Centromere-associated protein E isoform X1
wcw_0974	*Arabidopsis thaliana (thale cress)*	27.34	Viridiplantae	Streptophyta	Protein phosphatase 2C family protein
wcw_0982	*Thalassiosira pseudonana CCMP1335*	38.74	-	Bacillariophyta	Predicted protein
wcw_1002	*Dictyostelium purpureum*	22.57	-	-	Hypothetical protein DICPUDRAFT_53443
wcw_1052	*Medicago truncatula (barrel medic)*	17.84	Viridiplantae	Streptophyta	Hypothetical protein MTR_2g090985
wcw_1075	*Dictyostelium purpureum*	23.59	-	-	Hypothetical protein DICPUDRAFT_157374
wcw_1076	*Dictyostelium purpureum*	22.89	-	-	Hypothetical protein DICPUDRAFT_157374
wcw_1104	*Kryptolebias marmoratus (mangrove rivulus)*	21.03	Metazoa	Chordata	Golgin subfamily A member 4 isoform X1
**wcw_1131**	***Pediculus humanus corporis (human body louse)***	**21.35**	**Metazoa**	**Arthropoda**	**Conserved hypothetical protein**
wcw_1556	*Setaria italica (foxtail millet)*	20.6	Viridiplantae	Streptophyta	Uncharacterized protein LOC101755121, partial
wcw_1577	*Monodelphis domestica (gray short-tailed opossum)*	21.45	Metazoa	Chordata	CDK5 regulatory subunit-associated protein 2 isoform X1
wcw_1926	*Sorghum bicolor (sorghum)*	43.06	Viridiplantae	Streptophyta	Hypothetical protein SORBIDRAFT_01g043980

Supplementary Table S2: *W. chondrophila*-specific proteins with a Best Blast Hit against eukaryotic proteins.

Locus tag	Size (bp)	Function	Domain	SignalP/TMHMM
wcw_0138	1536	Hypothetical protein		
wcw_0521	1041	Choloylglycine hydrolase		
wcw_0538	633	Isochorismatase		
**wcw_1131**	**1941**	**Hypothetical protein**	**RasGEF domain**	
wcw_1238	1632	Hypothetical protein		Hydrophobic bi-lobed
wcw_1513	1257	Hypothetical protein		
wcw_1553	366	Hypothetical protein	Putative nucleotidyltransferase substrate binding domain	
wcw_1569	3807	Hypothetical protein		
wcw_1601	3540	Hypothetical protein		

Supplementary Table S3: Putative *W. chondrophila* T3SS effectors predicted *in silico* by three algorithms: BPBAac, Effective T3 and Modlab.

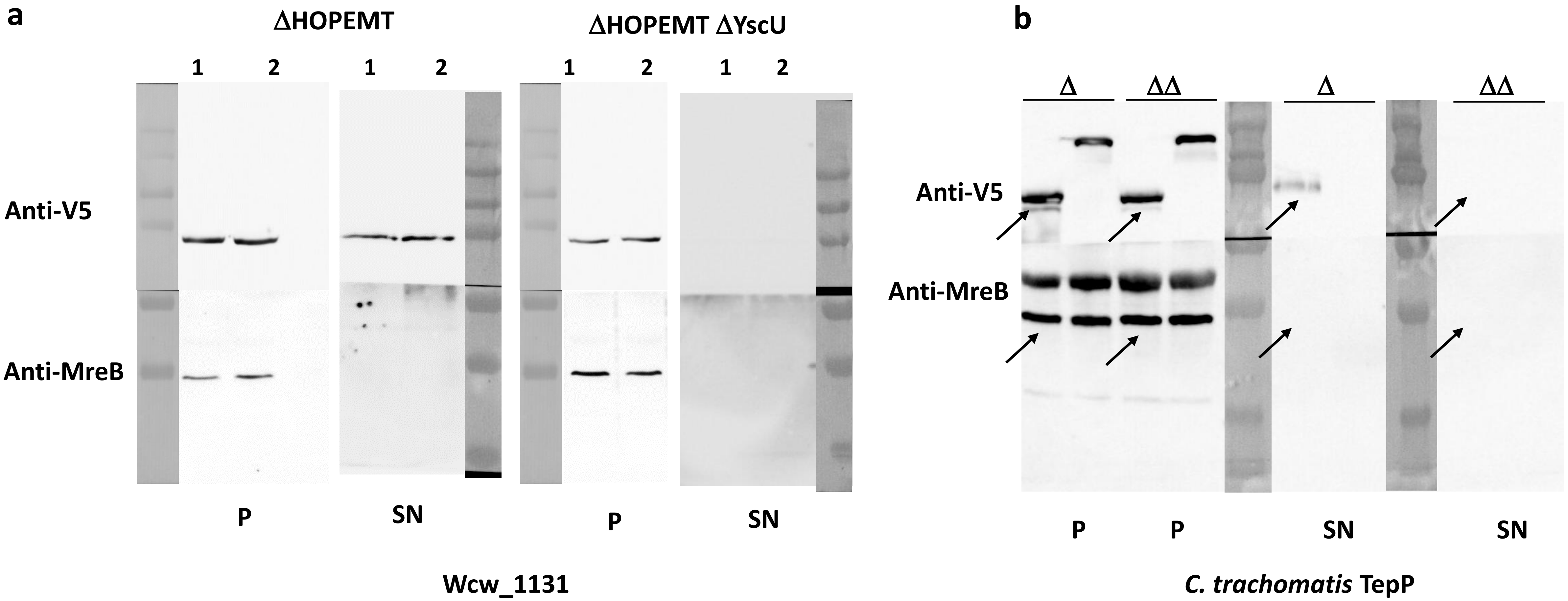

Supplementary Fig. S1: Full length immunoblots of secretion assays in *Y. enterocolitica* ΔHOPEMT and ΔHOPEMT ΔYscU strains. Epitope V5-tagged Wcw_1131 or *C. trachomatis* TepP (positive control) were detected in the bacterial pellet (P) or in the culture supernatant (SN). Arrows indicate bands that are presented in Figure 2.

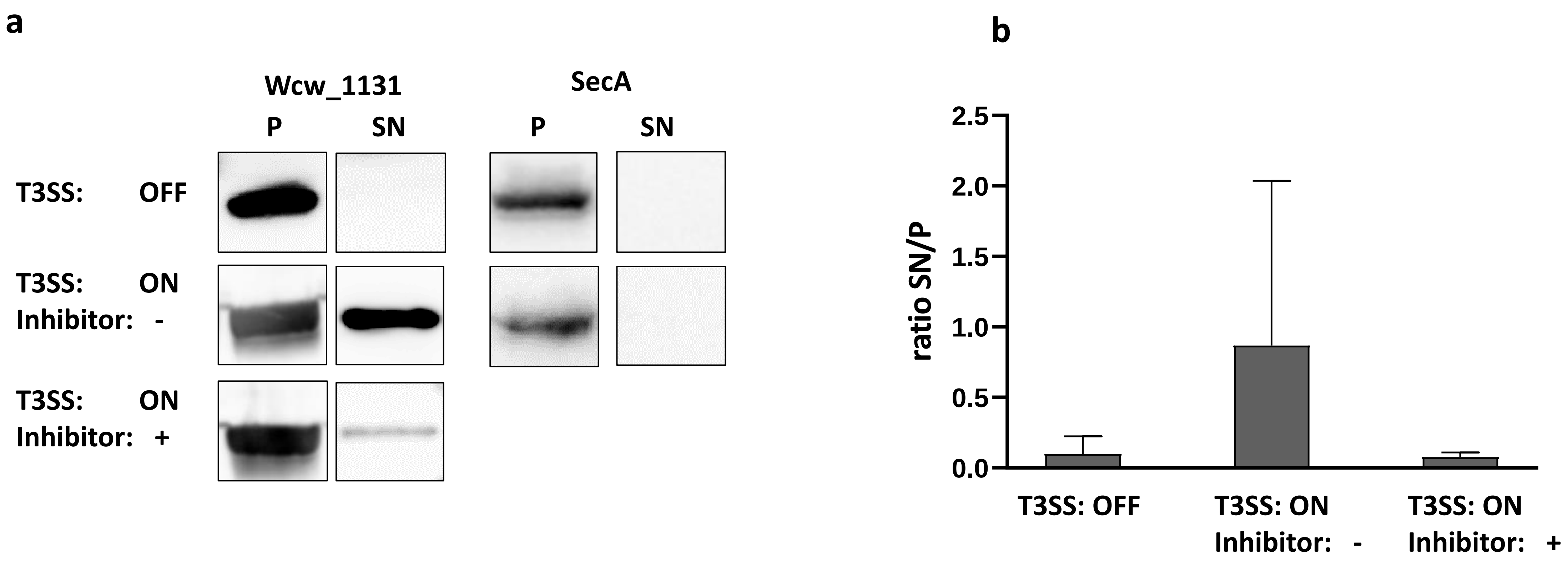

Supplementary Fig. S2: (a) Wcw_1131 and SecA (negative control) were detected by immunoblot in the bacterial pellet (P) or in the culture supernatant (SN) of *Y. enterocolitica* grown in presence (T3SS OFF) or in absence (T3SS ON) of calcium. A T3SS-specific inhibitor was added (inhibitor +) or not (inhibitor -) to the culture medium in absence of calcium (full length immunoblots are presented in Supplementary Figures S3). **(b)** Ratio of signal intensity detected in the supernatant versus pellet fractions as measured with Image J software. Results are the means and SD of at least three independent experiments.

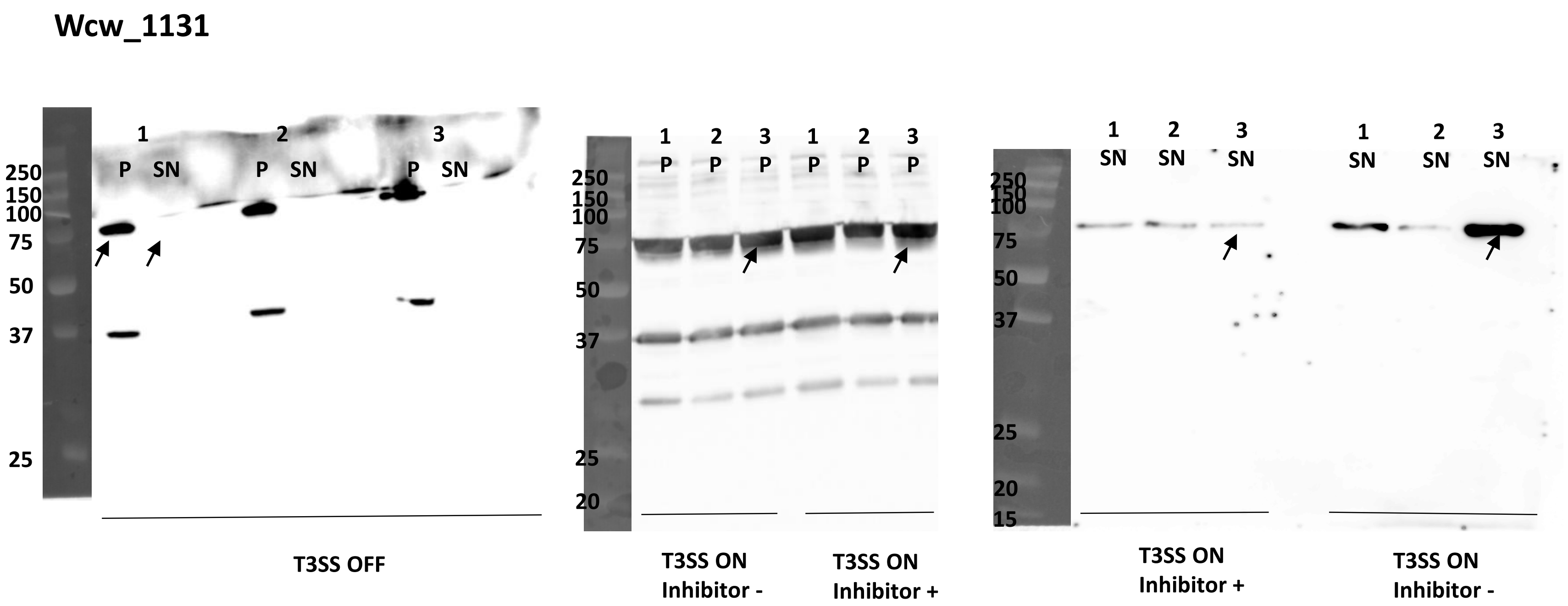

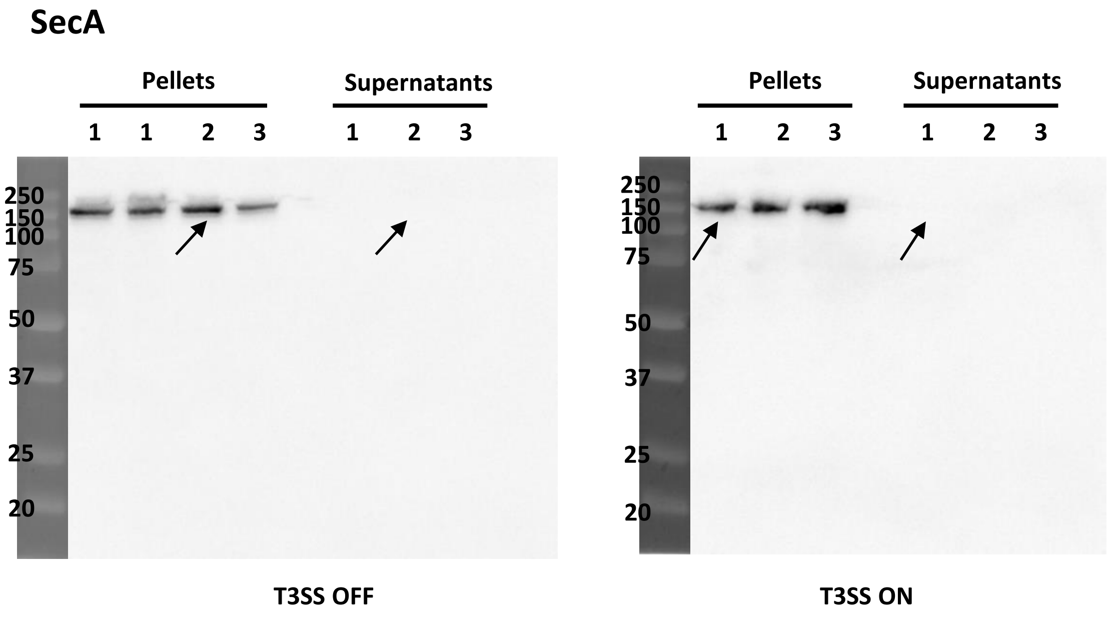

Supplementary Fig. S3: Full length immunoblots of secretion assays in *Y. enterocolitica* presented in supplementary Fig. 2. Wcw_1131 was detected in the bacterial pellet (P) or in the culture supernatant (SN) of *Y. enterocolitica* grown in presence (T3SS OFF) or in absence (T3SS ON) of calcium. A T3SS-specific inhibitor was added (inhibitor +) or not (inhibitor -) to the culture medium in absence of calcium. Experiments were performed in triplicates. Arrows indicate bands that are presented in Supplementary Fig. S2.

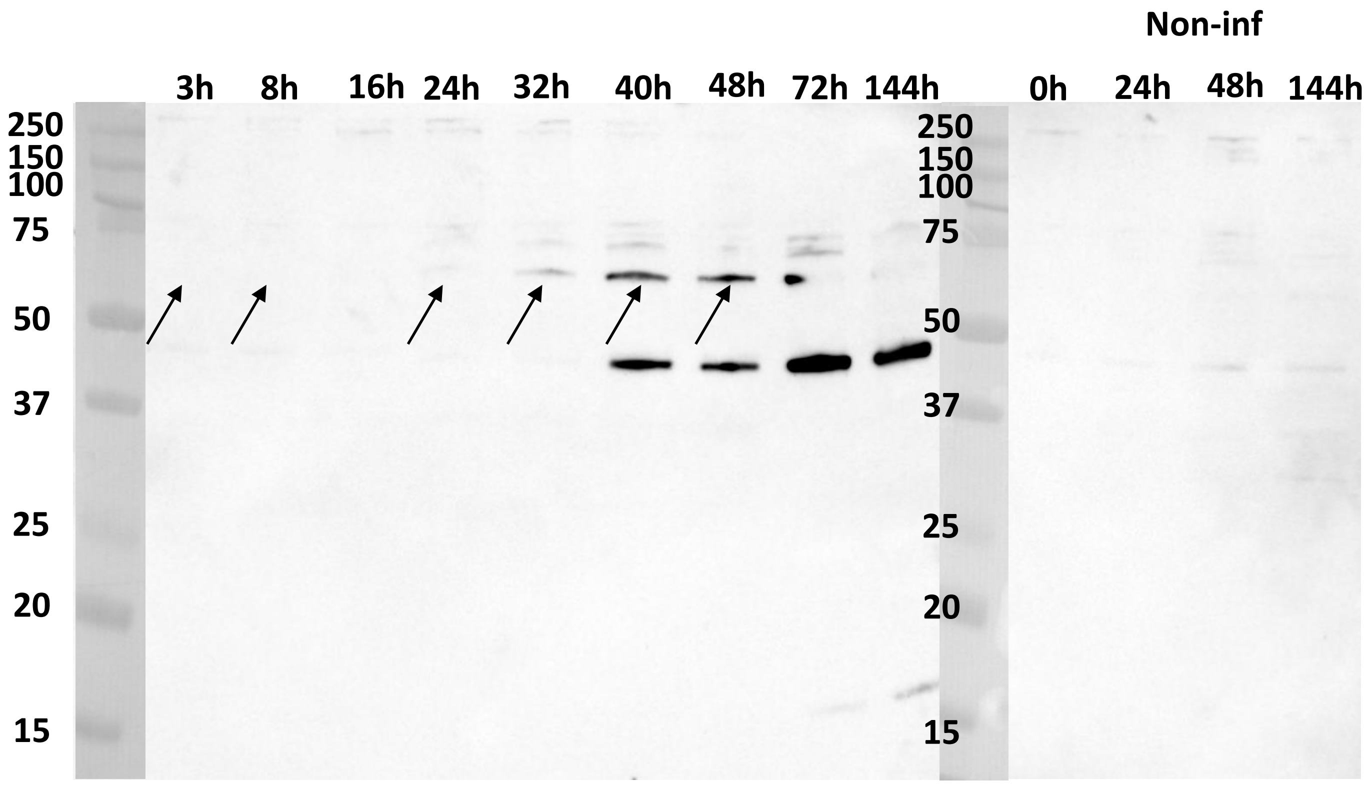

Supplementary Fig. S4: Full length immunoblot of Wcw_1131 protein expression at different time points during the course of *W. chondrophila* infection in Vero cells. Arrows indicate bands that are presented in Figure 3b.
